# Are the recommended proportions of mature body weight being achieved in different stages of growth in Brazilian Holstein and Jersey dairy cattle?

**DOI:** 10.3168/jdsc.2025-0743

**Published:** 2025-05-12

**Authors:** Marcos Busanello, Maihury Corrêa Santo, Rodrigo de Almeida

**Affiliations:** 1 Department of Animal Science, Federal University of Paraná, Curitiba, Paraná, 80035-050, Brazil; 2 Department of Animal Science, “Luiz de Queiroz” College of Agriculture, University of São Paulo, Piracicaba, São Paulo, 13418-900, Brazil; 3 Frísia Cooperativa Agroindustrial, Carambeí, Paraná, 84145-000, Brazil

## Abstract

The relationship between live BW (LBW) and mature BW (MBW) is critical for formulating optimal diets in dairy cattle, particularly regarding target BW (TBW) growth. Current nutritional guidelines suggest dairy cows reach MBW by the third calving (based on Holstein data), whereas beef cows reach it by the fourth calving. This study investigated LBW variations among different parities in Holstein and Jersey lactating cows, assessing the applicability of established proportion of MBW (%MBW) values across breeds. An observational study was conducted in dairy herds in Paraná State, Southern Brazil, comprising 19 Holstein and 9 Jersey herds, collecting LBW data at various growth stages. Nonlinear mixed models indicated MBW values of 705 kg for Holsteins and 460 kg for Jerseys, with Jersey cows showing faster maturation rate. Our findings revealed both breeds achieved recommended TBW verified by the %MBW at critical growth stages, but primiparous Jerseys exceeded the recommended values at first calving. Notably, while Holstein cows reach MBW by their third lactation, Jersey cows seem to achieve it by the fourth lactation, similar to beef cattle. This highlights the need for revised nutritional standards that better reflect the growth potential of different dairy breeds.

## Highlights

• MBW was estimated as 705 kg for Holstein and 460 kg for Jersey Brazilian cows.
• Both breeds are within the recommended TBW growth in younger life stages (2, 6, and 13-15 months of age).
• Brazilian Holstein cows reached the MBW in the third calving.
• Brazilian Jersey cows reached the MBW in the fourth calving.

## Graphical Abstract

**Figure:**
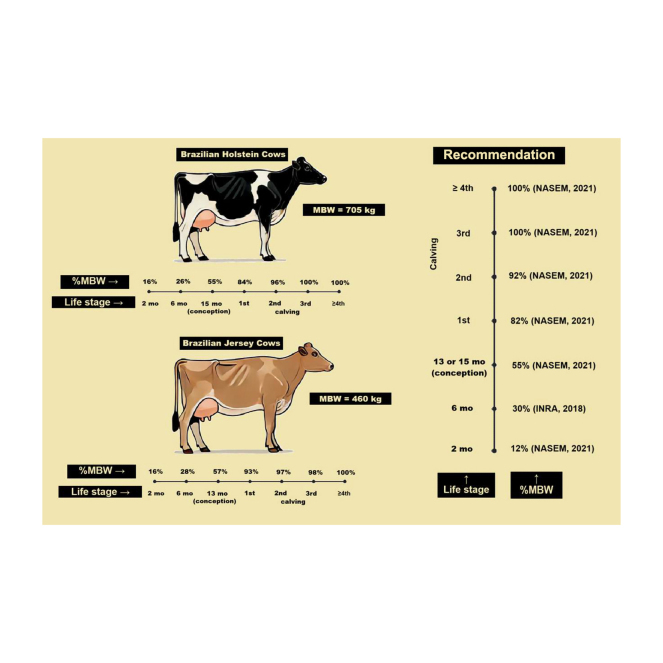
**Summary:** This study explored the relationship between live body weight (LBW) and mature body weight (MBW) in dairy cattle, focusing on Holstein and Jersey breeds. The research involved collecting LBW data across different cattle growth stages from 19 Holstein and 9 Jersey herds from Paraná State, Southern Brazil. Using nonlinear mixed models, the estimated MBW was 705 kg for Holsteins and 460 kg for Jerseys, with Jersey cattle maturing faster. Findings indicated both breeds met target BW (TBW) at critical growth stages, but primiparous Jerseys exceeded the recommended %MBW at first calving. While Holstein cows reached MBW by the third lactation, Jerseys achieved it by the fourth calving, similar to beef cattle. This suggests that current nutritional standards, currently based on Holstein data, need to be revised to accurately reflect the growth patterns of different breeds.

Currently, most of the nutritional requirement systems for dairy cattle use the relationship between live BW (**LBW**) and mature BW (**MBW**) to establish target BW (**TBW**) growth (NRC, 2001; INRA, 2007, 2018; CSIRO, 2007; NASEM, 2021). Studies suggest that lactating dairy cows typically reach their MBW by the third or fourth lactation (Fox et al., 1999; NRC, 2001; NASEM, 2021). This evidence emerged from studies with beef cattle, when the concept of TBW was proposed (Fox et al., 1992; NRC, 1996). The NRC Beef Cattle (NRC, 1996, 2000), based on data from Fox et al. (1992), suggested that beef cows continue to grow until their fourth calving, indicating differences in LBW among cows with different parities. From there, the proportion of MBW (%MBW) was used to estimate the TBW not only for the first conception but also for the first, second, third, and fourth calvings.

Fox et al. (1999), based on NRC Beef Cattle (NRC, 1996), used %MBW to estimate TBW in dairy cows, assuming growth until the fourth calving. The NRC Dairy Cattle (NRC, 2001) revised %MBW values based on Fox et al. (1999) but assumed growth only until the third calving, without clear justification (which was retained in NASEM, 2021). No studies have confirmed if dairy cows grow until their third or fourth calving under proper feeding. Additionally, these original studies focused on Holsteins (Fox et al., 1999; NRC, 2001), leaving the applicability to other breeds, such as Jersey, uncertain.

Holsteins are the most commonly reared dairy breed worldwide, mainly due to their high milk production. Conversely, Jerseys have seen increasing popularity in major milk-producing countries such as the United States (Olthof et al., 2023) and Canada (Wielen et al., 2020). In Brazil, the Southern and Southeast regions have the highest concentration of Jerseys (Martins et al., 2018). Jerseys, known for their smaller size, earlier maturity, and precocity (Busanello et al., 2022), may differ significantly in LBW growth compared with larger breeds such as Holsteins. This raises the question of whether the %MBW values proposed by nutritional systems, originally based on Holstein data, are appropriate for smaller-framed breeds like Jerseys, and whether these targets are actually achieved under practical conditions for both breeds in a Brazilian scenario.

A study with Brazilian dairy heifers (Jersey, Holstein, and Holstein × Gyr) found they reach ∼55% of their MBW at 13, 15, and 17 mo, respectively (Busanello et al., 2022), aligning with INRA (2018) and NASEM (2021) recommendations. However, %MBW for lactating cows was not assessed. Our study aims to evaluate LBW variations in Holstein and Jersey lactating cows, develop growth models, and compare predicted LBW with recommended %MBW to determine if they are met under practical conditions. We hypothesize that the recommended %MBW may not apply equally to both breeds.

This study was approved by the Animal Care Ethics Committee of the “Luiz de Queiroz” College of Agriculture, University of São Paulo (protocol number 2019-15) and was designed as a cross-sectional study. Holstein and Jersey dairy herds from Castro (24°47′32″S; 50°0′42″W; 996 m above sea level) and Carambeí (24° 56′ 59″S; 50° 6′ 35″W; 1,038 m above sea level) counties in Paraná State, Southern Brazil, were sampled. All cow and heifer data were collected at 3 time points: January to August 2017, August to October 2018, and November 2020, using a convenience sampling approach, with some farms measured across both time points.

The dataset included 19 Holstein herds (n = 576 cows and 2,034 heifers) and 9 Jersey herds (n = 368 cows and 860 heifers). Heifers were initially raised at a rearing center in Carambeí county, where measurements were taken at 2 time points. However, the rearing center decided to fully close its activities in August 2023. So, since 2019, some farmers began sending fewer heifers to the center, and as a result, the heifers were subsequently raised on their respective dairy farms. Heifer LBW was assessed up to 3 times from 3 mo of age onward, while cows were measured once. A quota sampling method was used, ensuring 10% to 20% of cows in each herd were measured to represent different parities.

In the studied farms, suckling calves were typically reared for 75 to 90 d before weaning. On the farms, most heifers were kept in confinement, with some on pasture and supplemented with concentrate. The farms' heifer-rearing systems varied depending on their life stages, including pasture, semi-confinement, and confinement, whereas heifers at the rearing center were exclusively confined. Feed management varied from farm to farm and season, with common pastures including *Cynodon dactylon*, ryegrass, wheat, and white oats. Supplemental feeds included hay, pre-dried forages, and silages, with some commercial concentrates provided, though their composition was not specified. Cows were raised in confinement systems, and all diets were adjusted according to NRC Dairy Cattle (NRC, 2001) and animal requirements.

The LBW was determined using a metric weighing scale tape (**WST**, Bovitec Produtos Agropecuários Ldta., São Paulo, SP, Brazil; Heinrichs et al., 1992; Wangchuk et al., 2018). The WST is designed for dairy cattle and includes 3 lines for different frame sizes: large (Holstein and Brown Swiss), medium (Ayrshire), and small (Jersey). Each line provides LBW values based on heart girth measurements. The large breed line was used for Holsteins and the small breed line for Jerseys. For accurate measurements, cattle were positioned standing with heads raised and facing forward, with the tape encircling the body behind the front legs without being too tight. Both heifers and cows were weighed in the morning and early afternoon.

Analyses were performed using SAS OnDemand (SAS Institute Inc., 2015). Descriptive statistics were conducted with PROC FREQ and PROC MEANS. PROC MIXED was used for LBW comparisons across parities, with Tukey–Kramer post hoc test. Nonlinear mixed models were fitted using PROC NLMIXED. PROC UNIVARIATE checked outliers and residual normality, PROC GLM conducted boxplots, and PROC REG assessed residual independence. A significance level of 0.05 was used.

Initially, descriptive statistics were performed to verify the coherence of the data for DIM, LBW, and age. Cows with different parities were grouped as follows: primiparous (first-calved cows), second-lactation cows, third-lactation cows, and fourth-or-greater-lactation cows, for both Holstein and Jersey cows. Mixed linear models were used to verify the differences for LBW among parities for both breeds. The general model was as follows:

$$y_{ijklm}=\mu +PO_{i}+DIM_{j}+age_{k}+herd_{l}+\varepsilon_{ijklm},$$

where *y_ijklm_* = LBW for the *i*th parity, *j*th DIM, *k*th age, *l*th cow in the *m*th herd; *µ* = intercept; *PO_i_* = fixed effect of parity order; *DIM_j_* = fixed covariate effect of DIM; *age_k_* = fixed covariate effect of age in months; *herd_l_* = random effect of repeated measures for the *m*th herd; and *ε_ijklm_* = random error associated to the observation *y_ijklm_*.

The random effect of repeated measures for the herd was tested using various variance-covariance matrices, and the final model selection was based on the lowest Akaike information criterion (**AIC**). Both models were assessed for the assumptions of residual normality, homogeneity, and independence. Residual normality was examined through quantile-quantile plots and Shapiro–Wilk's test, homogeneity was assessed using residuals' boxplots, and independence was evaluated through a graph of residuals plotted against model-predicted values. Outliers were verified using boxplots. No violations of these assumptions were detected.

Next, we conducted nonlinear mixed modeling for the LBW of both breeds. A dataset containing LBW of growing heifers was incorporated into the cow data to model the growth curves. Several nonlinear models, including Brody, Gompertz, logistic, and Von Bertalanffy, were fitted to the LBW data for Holstein and Jersey cattle. The logistic model demonstrated superior performance for both breeds based on fit statistics (AIC, R^2^, and root mean squared error) and it was consequently the selected one. The effect of repeated measures for the herd was included in the model. The general nonlinear logistic mixed model used was as follows:

y=[A/A(1+B×e(−K×age))(1+B×e(−K×age))]+δ+ε,

where *y* = LBW (kg); *A* = asymptote (related to MBW); *B* = coefficient of integration; *K* = maturation rate; *δ* = random effect repeated measures for the herds; and *ε* = random error.

The %MBW was calculated for both Holstein and Jersey cattle at some stages of their growth: weaning (2 mo of age), 6 mo, and conception (15 mo for Holstein and 13 mo for Jersey). For lactating dairy cows, the LBW considered for such a calculation was the median one for each parity. The MBW considered for calculations was 705 kg for Holstein and 460 kg for Jersey, obtained from the nonlinear mixed modeling of LBW growth. The calculation of %MBW was as follows:

$$\%MBW=100\times \left(\frac{LBW}{MBW}\right),$$

where %*MBW* = % of mature live body weight; *LBW* = live body weight at different growing stages; and *MBW* = mature body weight (Holstein = 705 kg, Jersey = 460 kg).

For Holstein cows, significant LBW differences were found among parities (*P* < 0.0001; Table 1). Primiparous cows had the lowest LBW (606 kg), followed by second-lactation cows (655 kg), whereas third-lactation (679 kg) and fourth-or-greater-lactation cows (684 kg) did not differ. For Jersey cows, all parities differed in LBW (*P* < 0.0001) (Table 1). Primiparous cows had the lowest LBW (410 kg), followed by second-lactation cows (428 kg), third-lactation (446 kg), and fourth-or-greater-lactation cows (475 kg).

**Table 1:** Adjusted means comparisons for live BW (LBW) from linear mixed models, considering different parity orders for Holstein and Jersey lactating dairy cows

Parity order^1^	Adjusted mean LBW^2^ (kg)	SEM	*P*-value
Holstein cows			<0.0001
Primiparous	606^C^	11.6	
2nd Lac cows	655^B^	10.3	
3rd Lac cows	679^A^	10.9	
≥4th Lac cows	684^A^	15.2	
Jersey cows			<0.0001
Primiparous	410^D^	12.0	
2nd Lac cows	428^C^	11.8	
3rd Lac cows	446^B^	11.8	
≥4th Lac cows	475^A^	12.2	

A–D Different superscript letters in columns for the parity orders of the same breed indicate significant differences at a 5% probability.

1 2nd Lac cows = cows in their second lactation; 3rd Lac cows = cows in their third lactation; ≥4th Lac cows = cows in their fourth-or-greater lactation.

2 Live BW.

The LBW of heifers was included in nonlinear growth modeling with cow data for both breeds to estimate MBW and maturity rate (%). The models predicted 705 kg MBW for Holsteins and 460 kg for Jerseys (Figure 1). Jersey cows had an earlier maturity rate (0.174% per month) compared with Holsteins (0.138% per month; Figure 1). These models were used to predict LBW at key growth stages (at weaning age – 2 mo, 6 mo of age, conception age – 15 mo for Holstein and 13 mo for Jersey) for calculating %MBW, while using the median LBW for lactating cows at each parity. A comparison of recommended TBW values indicated that the Holstein and Jersey cows in our study exhibited appropriate growth patterns to optimize milk yield, as shown in Table 2.

**Figure 1:**
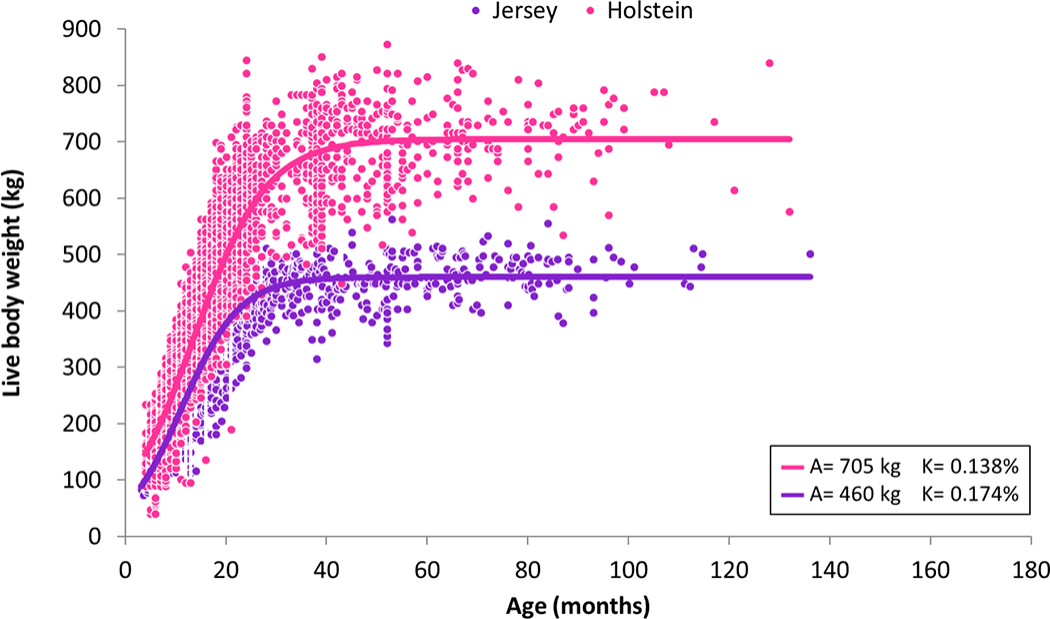
Live BW (kg) growth curves for Holstein and Jersey cattle regarding age (mo). Holstein: y=704.75/704.75(1+6.596×e(−0.1382×age))(1+6.596×e(−0.1382×age)), R^2^ = 0.87, root mean square error (RMSE) = 57.791; Jersey: y=460.14/460.14(1+7.181×e(−0.1741×age))(1+7.181×e(−0.1741×age)), R^2^ = 0.86, RMSE = 39.991. A = asymptote (related to MBW), and K = maturation rate.

**Table 2:** Proportion of mature BW (MBW) for Holstein and Jersey cows and heifers in our study, compared with literature values

Breed	Category^1^	LBW^2^ (kg)	Age (mo) for heifers and DIM^3^ (d) for cows	%MBW					
				Our study^4^	NRC Beef Cattle (NRC, 1996, 2016)	Fox et al. (1999)	NRC Dairy Cattle (NRC, 2001)	INRA (2007, 2018)	NASEM (2021)
Holstein	Weaning (2 mo)	117	2	0.16	—	—	—	—	0.12
	6 mo of age	182	6	0.26	—	—	—	0.30^5^	—
	Conception	385	15	0.55	0.55	0.55	0.55	0.50 to 0.60	0.55
	Primiparous	592	142	0.84	0.80	0.85	0.82	0.80	0.82
	2nd Lac cows	674	121	0.96	0.92	0.92	0.92	—	0.92
	3rd Lac cows	692	126	0.98	0.96	0.96	1.00	—	1.00
	≥4th Lac cows	716	130	1.02	1.00	1.00	1.00	—	1.00
	MBW	705	—	1.00	—	—	—	—	—
Jersey	Weaning (2 mo)	76	2	0.16	—	—	—	—	0.12
	6 mo of age	130	6	0.28	—	—	—	0.30^5^	—
	Conception	263	13	0.57	0.55	0.55	0.55	0.50 to 0.60	0.55
	Primiparous	427	119	0.93	0.80	0.85	0.82	0.80	0.82
	2nd Lac cows	444	110	0.97	0.92	0.92	0.92	—	0.92
	3rd Lac cows	449	95	0.98	0.96	0.96	1.00	—	1.00
	≥4th Lac cows	477	114	1.04	1.00	1.00	1.00	—	1.00
	MBW	460	—	1.00	—	—	—	—	—

1 2nd Lac cows = cows in their second lactation; 3rd Lac cows = cows in their third lactation; ≥4th Lac cows = cows in their fourth-or-greater lactation.

2 Live BW, being estimated for heifers using the nonlinear models, whereas for cows, it was the median LBW within each parity order group.

3 Days in milk, being the median DIM for each parity order group.

4 % of mature BW, which was obtained by the logistic nonlinear modeling.

5 INRA (2018, p. 307) model mentions that “the target weights of a dairy heifer are a little less than 30% of MBW at six months of age.”

The %MBW at weaning (2 mo; 16%) slightly exceeded the NASEM (2021) recommendation (12%) for both Holstein and Jersey cattle, possibly due to model overestimation since calves younger than 3 mo were not measured in our study. Since we extrapolated the model, the growth curve's behavior in this earlier life stage may differ. Possible causes of this overestimation could be differences in growth rates and physiological changes between preweaning (<2 mo) and weaned calves (>2 mo). The most likely scenario is that Holstein calves, for example, reach 117 kg of LBW at around 75 d of age, which is the typical median weaning age on those farms. At 6 mo, values were 26% for Holsteins and 28% for Jerseys, close to the INRA (2018) recommendation of “a little less than 30%” (Table 2). At conception, %MBW (∼55%) aligned with recommendations for both breeds (NRC, 1996, 2001; Fox et al., 1999; INRA, 2007, 2018; NASEM, 2021; Table 2). For lactating cows, the median LBW used for %MBW calculations corresponds to ∼4 mo of lactation, whereas literature typically refers to %MBW at calving. Primiparous Holsteins (84%) and second-lactation cows Holsteins (96%) met Fox et al. (1999) and NASEM (2021) recommendations (primiparous = 85%, second-lactation cows = 92%), whereas primiparous Jerseys (93%) exceeded recommendations (80%–85%). Second-lactation Jersey cows (97%) were within the recommended range. Third-lactation cows of both breeds (98%) had not yet reached MBW, suggesting MBW is likely attained by the fourth lactation, aligning more with Fox et al. (1999) than NRC (2001) or NASEM (2021). However, no differences were observed between third- and fourth-or-greater-lactation Holsteins (Table 1).

Findings reveal that %MBW under practical rearing conditions meet recommendations at weaning (2 mo), 6 mo, and conception, similar to Busanello et al. (2022) for %MBW at conception (Holstein: 58%, Jersey: 56%). However, our data showed lower estimated ADG from weaning (2 mo) to conception for Holsteins (691 vs. 844 g/d) and slightly higher for Jerseys (574 vs. 540 g/d). Conversely, estimates of ADG from conception to first calving was higher for both breeds in our study (Holstein: 670 vs. 534 g/d; Jersey: 554 vs. 480 g/d).

Literature suggests an ADG of 800 g/d during the growth phase for Holsteins (Zanton and Heinrichs, 2005) leading to 80% to 85% MBW at first calving (NRC, 1996, 2001, 2016; Fox et al., 1999; INRA, 2007, 2018; NASEM, 2021). Heifers with higher %MBW at first calving perform better in milk production and profitability (Handcock et al., 2018). In New Zealand, the target is 90% MBW (Handcock et al., 2019). For Brazilian dairy herds, Busanello et al. (2022) found %MBW for primiparous Holstein (80%) and Jersey cows (86%) aligned with NRC (2001) and NASEM (2021) recommendations. Differences in ADG between our findings and Busanello et al. (2022) may arise from distinct rearing practices and regional variations in cattle body frame, reflected in the 20 kg difference in MBW (705 vs. 681 kg for Holstein and 460 vs. 440 kg for Jersey).

The LBW and MBW are crucial for dietary formulation in dairy cattle, with most programs using breed-specific MBW values. NASEM (2021) includes 72 equations that require BW as input. The MBW determines TBW and growth monitoring in nutritional systems. Holstein MBW recommendations range from 600 kg (CSIRO, 2007) to 750 kg (INRA, 2018), with NASEM (2021) suggesting 700 kg. For Jerseys, values range from 400 kg (CSIRO, 2007) to 520 kg (NASEM, 2021). However, MBW varies within herds and breeds (Berry et al., 2005), highlighting the need for herd-specific measurements for accurate dietary formulations.

We observed LBW differences across parities for both lactating Holstein and Jersey cows. All parities differed, except third- versus fourth-or-greater-calving Holsteins, suggesting that Jerseys may continue to grow until the fourth calving, contradicting NRC Dairy Cattle (NRC, 2001) and NASEM (2021), which state cows reach MBW by the third calving. Results for Jerseys align more closely with NRC Beef Cattle (NRC, 1996, 2016) and Fox et al. (1999), which suggest MBW is reached by the fourth calving. However, our data reflect LBW at 3 to 4 mo of lactation, not postcalving. At this stage, cows are assumed to be beyond negative energy balance and in an intermediate BCS, similar to calving time (Poncheki et al., 2015).

Based on those findings a question arises: Why do NRC Dairy Cattle (NRC, 2001) and NASEM (2021) suggest cows reach MBW by the third calving? NASEM (2021) retained the %MBW recommendations from NRC Dairy Cattle (NRC, 2001), which were based on Van Amburgh et al. (1998) data that only used an MBW of 641 kg and did not evaluate LBW by parities. Among referenced NRC Dairy Cattle (NRC, 2001) studies (Hoffman, 1997, and Kertz et al., 1997, 1998), only Kertz et al. (1997) assessed LBW by parity, reporting an MBW of 730 kg for fourth-or-greater-lactation Holsteins. Their postcalving %MBW values (79% for primiparous, 87% for second-lactation cows, and 96% for third-lactation cows) align more with NRC Beef (NRC, 1996, 2016) and Fox et al. (1999) than NRC Dairy Cattle (NRC, 2001) and NASEM (2021). The NRC Dairy Cattle (NRC, 2001) did not fully evaluate %MBW across parities, despite evidence suggesting MBW is reached at the fourth calving.

An important point is that NRC Dairy Cattle (NRC, 2001) was based solely on Holstein cattle because studies by Hoffman (1997), Kertz et al. (1997, 1998), and Van Amburgh et al. (1998) only used Holsteins. Our results suggest that the same %MBW for setting TBW may apply to both Holstein and Jersey young cattle, but not to lactating growing cows. This supports our hypothesis that literature-recommended %MBW may not be equally applicable to both breeds, with similar findings for Holstein × Gyr cattle (Busanello et al., 2022).

However, our study has some limitations that should be acknowledged. Our sample size is limited to a few herds and does not include a large number of animals, which may restrict the generalizability of the results to other regions and warrants cautious interpretation. Dietary planning varies from farm to farm, differing in growth objectives, rearing systems, genetics, and feed types, but we attempted to account for this farm variability in our statistical modeling by including the “herd” effect as a random factor. The same issue about variability applies to MBW.

Finally, our findings suggest that Brazilian Holstein and Jersey growing cattle meet their TBW based on the recommended %MBW in the literature. Contrary to the common view that cows reach MBW by the third calving (NRC, 2021; NASEM, 2021), our study indicates that Brazilian Jersey cows reach MBW by the fourth calving, aligning with Fox et al. (1999) and NRC Beef Cattle (NRC, 1996, 2016). This indicates the need for further research on LBW and %MBW across parities, especially for breeds beyond Holstein and in different global regions.
